# When the Conception of Symmetry Deceives Us: A Case Report on the Perioperative Diagnosis of Subclavian Artery Stenosis

**DOI:** 10.7759/cureus.48699

**Published:** 2023-11-12

**Authors:** Mariana Adams, Cristina P Sousa, Sónia Duarte, Humberto Machado

**Affiliations:** 1 Department of Anesthesiology, Centro Hospitalar Universitário do Porto, Porto, PRT; 2 Department of Anesthesiology, Centro Hospitalar de Trás-os-Montes e Alto Douro, Vila Real, PRT; 3 Department of Anesthesiology, Instituto de Ciências Biomédicas Abel Salazar, Universidade do Porto, Porto, PRT

**Keywords:** pulse, physical examination, blood pressure, stenosis, anesthesia

## Abstract

Subclavian artery stenosis is a relatively uncommon manifestation of peripheral artery disease with significant morbidity. We describe a case of left subclavian artery stenosis that became apparent in the intraoperative setting, in a patient who presented for emergency surgery after a fall and subsequent left femoral neck fracture. Initial non-invasive blood pressure readings on the left upper extremity were in the normal range but after placement of the arterial line on the right upper extremity, the discrepancy was evident pointing towards a structural abnormality as the causative factor. CT angiography was found to confirm the diagnosis of left subclavian artery stenosis of an atherosclerotic nature. A thorough physical examination can point to the presence of subclavian stenosis through pulse amplitude and blood pressure asymmetries. This study highlights the importance of obtaining blood pressure readings ideally on all four limbs to avoid misinterpretation of blood pressure values that could ultimately lead to the use of unnecessary and inadequate interventions and possible complications in the perioperative setting.

## Introduction

Subclavian artery stenosis is a form of peripheral artery disease (PAD) whose presence causes notable comorbidity, with ischemic manifestations ranging from limb claudication to cerebral hypoperfusion and coronary steal syndrome [1]. A physical examination can point to the diagnosis through asymmetry in upper limb peripheral pulses and a systolic difference of more than 15 mmHg contralaterally. Traditional risk factors for PAD are usually present such as smoking, hypertension, diabetes mellitus, and hyperlipidemia. The presence of this condition can lead to erroneously low blood pressure recordings in the affected limb presenting an anesthetic challenge if the diagnosis is not previously known or is not considered. We present a case of unilateral subclavian stenosis in a patient undergoing surgical management of a femoral neck fracture in the emergency setting. 

This article was previously presented as a poster at the 2023 Portuguese Society of Anesthesiology Annual Congress on March 25, 2023.

## Case presentation

A 75-year-old female, independent and living in her own home without assistance, was admitted to the emergency department after a fall resulting in a fracture of the left femoral neck. Her past medical history included hypertension, non-insulin-dependent diabetes mellitus, class 1 obesity, paroxysmal atrial fibrillation, significant ischemic heart disease, and pulmonary hypertension. Her chronic medication included anticoagulation with apixaban, antiaggregation with acetylsalicylic acid, lisinopril, bisoprolol, and amiodarone. Her baseline exercise tolerance was <4 metabolic equivalents (METs), and she complained of dyspnea and fatigue with ordinary physical activity. She was deemed American Society of Anesthesiologists-Physical Status 4 (ASA-PS 4).

The patient had a coronary artery bypass graft procedure performed in 2004 and was discharged from outpatient consultation in 2006. She was readmitted in 2015 due to persistent angina pectoris. The coronary angiography performed demonstrated disease of the native vessels with subocclusive stenosis of the left coronary artery and a dominant right coronary artery with critical stenoses in the proximal and medial segments. The patient was placed on optimal medical therapy as the risks of a percutaneous coronary intervention to treat the right coronary artery were deemed to be high. The patient developed an iatrogenic arteriovenous femoral fistula as a complication of the coronary angiography performed and was treated with an endovascular procedure in July 2022.

At the beginning of 2022, the patient was diagnosed with pulmonary embolism after presenting to the emergency department with chest pain and dyspnea. In the outpatient clinic, the study for chronic thromboembolic pulmonary hypertension was completed with a transthoracic echocardiogram, ventilation-perfusion scan, and right heart catheterization. The ventilation-perfusion (V/Q) scan showed no persistent deficits and the right heart catheterization demonstrated a hemodynamic profile compatible with group 2 pulmonary hypertension.

**Figure 1:**
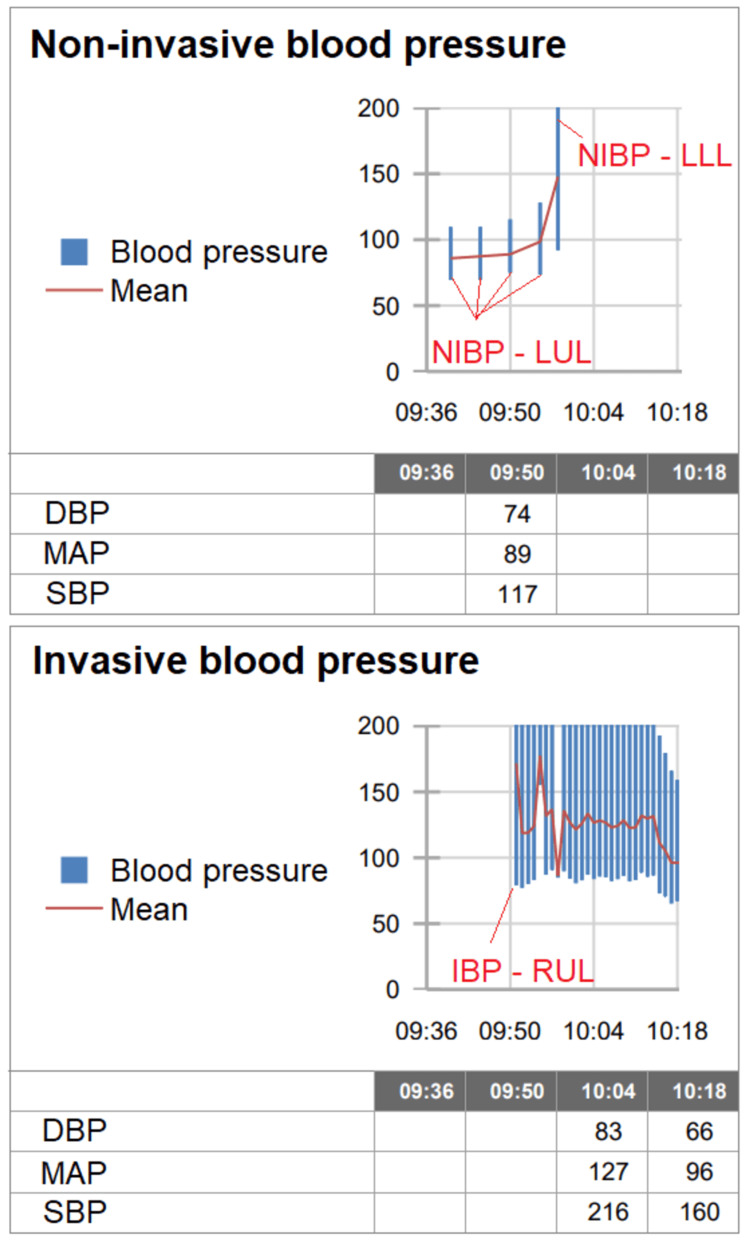
Graph of BP and heart rate tendencies in the operating theater. NIBP-LUL: non-invasive blood pressure reading on the left upper limb; IBP-RUL: invasive blood pressure monitoring in the right upper limb through a radial arterial line; NIBP-LLL: non-invasive blood pressure reading on the left lower limb; DBP: diastolic blood pressure; MAP: mean arterial pressure; SBP: systolic blood pressure

The anesthesia team on call was informed of the patient’s diagnosis and proposed surgical intervention. The patient denied any history of anesthetic complications. Temperature, heart rate, peripheral oxygen saturation, and blood pressure (BP) measurements in the ward were stable with the highest BP recorded value of 123/76 mmHg. Informed consent was obtained for surgery and for general anesthesia with a peripheral nerve block to optimize post-operative analgesia. In the operating room, the patient was monitored according to ASA standards, invasive BP with an arterial line, frontal cerebral near-infrared spectroscopy (NIRS) (O3 Regional Oximetry System; Irvine, CA: Masimo), processed EEG (SedLine; Irvine, CA: Masimo), and hourly urine output. The non-invasive BP cuff was placed on the left upper arm with a recorded blood pressure reading of 117/74 mmHg. With the patient in dorsal decubitus, we proceeded with an ultrasound-guided lateral femoral cutaneous nerve of the thigh and femoral nerve block with a total of 40 mL of ropivacaine at 0.25%. Because of the cardiovascular risk factors of the patient and the history of pulmonary hypertension and chronic pulmonary embolism, we decided that an arterial line was warranted to closely monitor changes in blood pressure and allow for arterial blood gas sampling. When palpating the peripheral pulses, the left radial pulse was noted to be weak when compared to the contralateral limb. After the placement of the catheter on the right radial artery, the blood pressure values obtained were 216/83 mmHg. BP readings on the lower limbs coincided with the arterial line values on the right radial artery (Figure 1). This discrepancy in blood pressure readings combined with the asymmetry in pulse intensity led us to consider the possibility of a structural cause, such as arterial stenosis. General anesthesia was induced with a bolus injection of fentanyl (1.5 μg/kg) followed by a bolus injection of propofol (1 mg/kg). The patient was curarized with rocuronium and tracheal intubation was successful with direct laryngoscopy. We opted for total intravenous anesthesia with continuous infusion of propofol at 50 μg/kg/min and subsequently adjusted the rate according to changes in the processed EEG with a target patient state index (PSI) range between 25 and 50. The patient was mechanically ventilated with a low tidal volume strategy of 6 mL/kg.

Before incision, antibiotic prophylaxis with cefazolin 2 g IV was administered. Hemodynamic stability was observed throughout most of the procedure with arterial blood pressure lowering after induction to a mean arterial pressure (MAP) of about 95-100 mmHg. A drop in blood pressure was observed at about 1 h 15 min into the 2 h long procedure that corresponded to the time of cementation in the surgical procedure, with a drop in BP to 94/45 mmHg (62 mmHg MAP). There was no associated drop in oxygen saturation or NIRS but due to the greater than 20% drop in systolic blood pressure, we considered this situation a grade 1 bone cement implantation syndrome [2].

Two 100 µg boluses of phenylephrine separated by a 5-min interval were administered with subsequent improvement of blood pressure values. Arterial blood gas samplings performed showed no major abnormalities. A 500 mL polyelectrolyte solution was administered at a slow rate throughout the procedure with 115 mL of urine output recorded and 300 mL of estimated blood loss.

The remaining procedure and immediate post-operative period in the post-anesthetic care unit occurred without complications. Analgesia was provided with paracetamol and tramadol. Deep vein thrombosis prophylaxis was instituted with compression stockings and enoxaparin at 40 mg/kg. In the intermediate care unit, the patient developed hypoxic and hypercapnic respiratory failure with atelectasis and obesity-hypoventilation syndrome as the suspected etiologies. Gas exchange improved after the introduction of non-invasive ventilation and the patient was discharged to the ward on the fifth post-operative day. On the sixth post-operative day, the patient presented a left-sided grade 4 hemiparesis and a CT angiography of the head and neck was obtained due to a suspected cerebral vascular accident of cardioembolic origin. Both the first CT scan and the repeat 24 h control scan were negative for recent vascular lesions. After discussion with the surgical team, the anticoagulant dose was increased to the therapeutic range. The patient began daily physical therapy in the ward and at the time of discharge on the seventeenth post-operative day, the patient had made a full recovery with no neurological deficits on examination.

**Figure 2:**
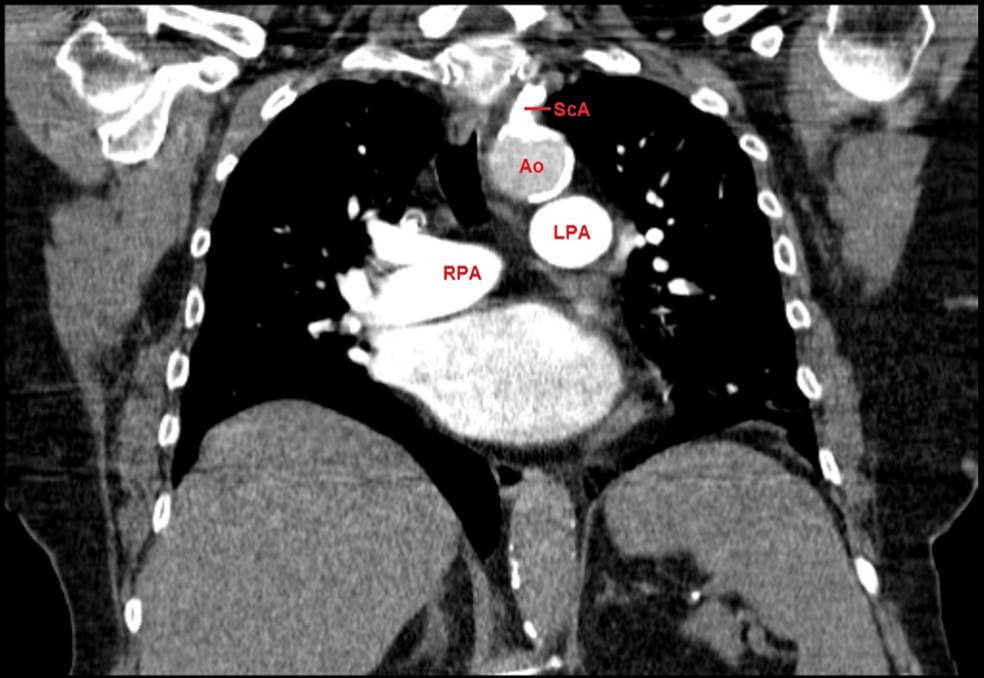
CT scan angiography of the supra-aortic arch: calcification visible at the emergence of the left subclavian artery. ScA: left subclavian artery; Ao: aorta; RPA: right pulmonary artery; LPA: left pulmonary artery

We then proceeded to investigate the patient’s electronic medical file as the first step in our diagnostic ladder and found a CT angiogram report of the chest and supra-aortic trunks requested in the outpatient setting by the patient’s attending cardiologist. The report described exuberant calcification at the origin of the left subclavian artery, with extension to the adjacent aortic wall (Figure 2). The left subclavian artery refills after the calcification but is thinner than the contralateral vessel, with irregularity in the transition between the subclavian and the axillary artery. This led us to consider that, in the ward, blood pressure readings were most likely taken consistently on her left upper arm which explains the values recorded. Because the values measured were consistently at a low-normal range, her blood pressure medication was not initiated which contributed to the markedly high values observed on placement of the arterial line on the right radial artery. This diagnosis was afterward highlighted in the anesthetic report as well as the patient’s medical file to avoid future misinterpretation of varying blood pressure readings.

## Discussion

Subclavian artery stenosis is a relatively uncommon manifestation of PAD with significant morbidity as its symptomatic manifestations can range from upper limb ischemia to cerebral hypoperfusion and coronary steal syndrome. It has a reported incidence between 0.5% and 2%, however, in patients with lower extremity arterial disease this percentage can be as high as 9% [3].

It has been described as a relevant marker of severity of PAD, where about 50% of patients with this diagnosis are also found to suffer from coronary artery disease and about 30% are at risk of carotid and vertebral artery disease [4].

Subclavian artery stenosis is asymptomatic in the majority of patients as it is a clinical entity characterized by slow disease progression. Symptoms tend to arise only when luminal diameter narrows to <50% [1]. Therefore, one must have a high degree of clinical suspicion as many patients are asymptomatic and will present only with asymmetry in blood pressure readings as seen in our patient.

The major cause of subclavian artery stenosis is atherosclerotic disease [5]. Risk factors for atherosclerosis include hypertension, diabetes mellitus, hyperlipidemia, and tobacco consumption. Our patient had three of these cardiovascular risk factors as well as obesity and a strong family history of ischemic heart disease. Other conditions to consider include congenital abnormalities as risk factors for stenosis, arteritis, such as Takayasu, radiation exposure and consequent inflammatory stenotic response, fibromuscular dysplasia, and external compression, such as in arterial thoracic outlet syndrome. 

Clinical manifestations vary according to anatomic factors, such as the degree of narrowing, the etiology of the stenosis, and the presence of arterial disease in other territories. The left subclavian artery is affected more often than the right or innominate arteries [6]. Though isolated subclavian artery stenosis is more likely to be asymptomatic, the presence of an obstruction in other vessels, such as the carotid artery, vertebral artery, or aortic arch vessels, increases the likelihood of symptomatic disease. Patients can present with upper extremity manifestations, such as hypoesthesia, claudication, muscle fatigue, rest pain, and tissue necrosis [1]. In vertebrobasilar hypoperfusion due to subclavian steal syndrome, complaints of vertigo, syncope, dysarthria, ataxia, and gaze abnormalities are common as blood flow is diverted from the posterior cerebral circulation to supply the arm during exertion [7,8]. A rarer manifestation of subclavian stenosis is the coronary subclavian steal syndrome that occurs in patients submitted to coronary artery bypass graft surgery. After left internal mammary artery (LIMA) grafting, proximal stenosis of the subclavian artery could lead to a reversal of flow from the LIMA to the subclavian vessel with subsequent cardiac hypoperfusion [9].

In unilateral stenosis, systolic pressure differences of >15 mmHg can be found as well as a fainter pulse on the affected side. In bilateral stenosis, measuring the blood pressure in the upper and lower limbs and noting an Ankle Brachial Index (ABI) value of >1.3 can help point to the diagnosis [10]. It is important to consider this diagnosis in cases of refractory hypotension after the exclusion of other more common etiologies of hypotension [11].

A thorough physical examination can point to the presence of subclavian stenosis if blood pressure measurements are taken from the four limbs. This study is meant to highlight the importance of this step in the perioperative setting to avoid misinterpretation of blood pressure values that could ultimately lead to the use of vasoactive drugs unnecessarily.

## Conclusions

This study aimed to highlight the importance of clinical integration between findings in the physical examination of the patient and discrepancies in blood pressure readings and to remind us that the ideal of symmetry is misleading. In retrospect, we gained an even greater appreciation for the importance of a thorough pre-anesthetic evaluation in the emergency setting, including not only a targeted physical examination but also a comprehensive examination of all available patient history. It is also important to document this finding clearly in the patient’s medical record and inform the team responsible for her care in the ward, as suboptimal treatment of hypertension can increase the risk of perioperative complications.

## References

[1] Caesar-Peterson S, Bishop MA, Qaja E (2023). Subclavian artery stenosis. StatPearls [Internet].

[2] Donaldson AJ, Thomson HE, Harper NJ, Kenny NW (2009). Bone cement implantation syndrome. Br J Anaesth.

[3] Aboyans V, Ricco JB, Bartelink ME (2018). 2017 ESC Guidelines on the diagnosis and treatment of peripheral arterial diseases, in collaboration with the European Society for Vascular Surgery (ESVS): document covering atherosclerotic disease of extracranial carotid and vertebral, mesenteric, renal, upper and lower extremity arteries. Eur Heart J.

[4] Jahic E, Avdagic H, Iveljic I, Krdzalic A (2019). Percutaneous transluminal angioplasty of subclavian artery lesions. Med Arch.

[5] Salman R, Hornsby J, Wright LJ, Elsaid T, Timmons G, Mudawi A, Bhattacharya V (2016). Treatment of subclavian artery stenosis: a case series. Int J Surg Case Rep.

[6] Bosiers M, Deloose K, Verbist J, Peeters P (2011). Subclavian and vertebral arteries: angioplasty and stents. Endovascular Surgery. Fourth Edition.

[7] Psillas G, Kekes G, Constantinidis J, Triaridis S, Vital V (2007). Subclavian steal syndrome: neurotological manifestations. Acta Otorhinolaryngol Ital.

[8] Pirau L, Lui F (2022). Vertebrobasilar insufficiency. StatPearls [Internet].

[9] Younus U, Abbott B, Narasimha D, Page BJ (2014). Coronary subclavian steal syndrome: an unusual cause of angina in a post-CABG patient. Case Rep Cardiol.

[10] Nasrullah A, Singh R, Hamza A, DiSilvio BE (2022). Pseudoshock: a challenging presentation of bilateral subclavian artery stenosis. Eur J Case Rep Intern Med.

[11] Toh MR, Lee D, Damodharan K, Abdullah MA (2019). Case report: an unusual presentation of bilateral subclavian stenosis in a patient with asymptomatic hypotension. Am J Case Rep.

